# Comprehensive RNA-Seq analysis revealed molecular pathways and genes associated with drought tolerance in *Morus alba* cv. Yunsang-2

**DOI:** 10.1186/s12870-026-09479-6

**Published:** 2026-07-15

**Authors:** Jie Li, Yi Deng, Qirong Ma, Weifen Liu, Xianyan Zou

**Affiliations:** College of Biology and Food Engineering, Qujing Normal University, Qujing, 655011 China

**Keywords:** Mulberry, Drought tolerance, RNA-Seq, Yunsang-2, Transcription factor

## Abstract

Drought stress driven by global climate change critically restricts mulberry growth. The identification of drought-responsive genes in the Yunnan-adapted Yunsang cultivar is essential for mitigating environmental constraints on sericulture. In this study, seedlings of the mulberry cultivar *Yunsang-2* were subjected to drought stress under greenhouse conditions. Leaf samples were collected for physiological analysis (proline content and CAT and POD activities) and transcriptome profiling via RNA-Seq. The results revealed that compared with the plants in the CK group, the drought-stressed plants had significantly increased CAT and POD activities by 7 days post-stress (DPS) and accumulated markedly greater amounts of proline at 9 and 12 DPS. Transcriptomic analysis revealed that drought resistance involves key genes enriched in the abscisic acid (ABA), gibberellin (GA), and brassinosteroid (BR) signaling pathways, such as PYR, ABF, PIF3, and BSK. Furthermore, we identified 156 TFs as potential regulatory hubs. Among these genes, *MnERF21* was tentatively identified as a candidate positive regulator of drought resistance. Our findings systematically elucidate the molecular mechanisms underlying drought tolerance in mulberry and provide novel insights into the drought resistance strategies of Yunnan-adapted germplasms.

**Supplementary Information:**

The online version contains supplementary material available at https://doi.org/10.1186/s12870-026-09479-6.

## Introduction

*Morus alba* L. (mulberry) is an economically important tree species with extensive cultivation across China and rich germplasm resources, boasting a cultivation history exceeding 5,000 years [1]. Both the leaves and the fruits of mulberry possess significant economic and medicinal value [2]. The leaves serve as the primary food source for silkworms. Analysis of phytochemical extracts revealed that mulberry leaves contain bioactive compounds such as flavonoids and alkaloids, which demonstrate hypoglycemic, hepatoprotective, and vision-improving effects [3, 4]. Similarly, mulberry fruits are rich in anthocyanins, vitamins, and minerals, contributing to their traditional use in nourishing blood, replenishing yin, and promoting bowel function [5, 6]. Consequently, expanding mulberry cultivation is economically and pharmaceutically desirable. However, the increasing frequency of extreme climate events, including floods and droughts driven by global climate change, poses a critical threat to mulberry productivity and associated economic value chains [7, 8]. Statistical data indicate that a strategic shift occurred in China’s sericulture industry from the central region to the western region in 2024 [9]. As the exclusive food source for silkworms, mulberry plantations are consequently relocating accordingly. Notably, within Yunnan Province, where numerous economically disadvantaged areas persist, sericulture activities (mulberry cultivation and silkworm rearing) constitute vital economic livelihoods for many farmers.

Southwest China experiences pronounced cyclical variations between distinct rainy (May to November) and dry (November to April) seasons [10], which results in a reduction in grass and crop yields due to water shortages. Therefore, genetic approaches for discovering potential genes and mechanisms of drought resistance are primary strategies for improving the efficiency of water and maintaining the life activity of mulberry.

In recent decades, many abiotic stress resistance pathways, such as ABA signaling and MAPK, and TFs, such as NAC, WRKY and ERFs, have been reported in the *Populus* and *Malus* genera [11–13]. Overexpression of *PePYL4* significantly increased ABA sensitivity and reduced the stomatal aperture to increase drought resistance in the *Populus* genus. In addition, the upregulated MAPK genes induced by arbuscular mycorrhizal fungi (AMF) could increase drought tolerance in *Populus simonii* [14]. Furthermore, epigenetic modifications of H3K9ac activated the expression of NAC TFs, including *PtrNAC006*, *PtrNAC007*, and *PtrNAC120*, for drought response and tolerance in *Populus* species [15]. In *Malus* species, *MdMYB44-like* enhances drought and salt tolerance via the MdPYL8-MdPP2CA module in ABA signaling [16]. *MdNAC29* negatively regulates drought resistance by directly repressing *MdDREB2A* expression in apple [17]. In summary, TFs and plant signal transduction pathways play crucial roles in the response to plant abiotic stress.

In *Morus* species, genes such as *MmSK*, *MnEIL1* and *RACK1* are related to drought resistance [18–24]. In addition, the application of omics technologies revealed that pathways such as ABA and flavonoid biosynthesis participate in enhancing the drought tolerance of mulberry [25–29]. However, current knowledge regarding drought-responsive genes and pathways in mulberry remains limited [30, 31].

Given that China has rich mulberry germplasm resources, mining hub genes from drought-tolerant cultivars that exhibit superior agronomic traits holds significant potential for expanding mulberry cultivation to marginal lands. In the present study, Yunsang-2, a putatively drought-tolerant mulberry cultivar released more than three decades ago that also exhibits superior fruit quality, including the highest contents of vitamin C, phenolics, and anthocyanins among the tested varieties [32], was selected for drought resistance gene screening through physiological measurements and RNA-Seq. This cultivar has been extensively planted in Yunnan Province and represents a valuable locally adapted germplasm.

On the basis of the contents of proline and soluble protein and the activity of CAT and POD, seedlings subjected to 7 and 12 days of drought treatment were subjected to RNA-Seq. Consequently, DEGs between the control (CK) and drought-stress (DS) groups were identified. Venn diagram analysis was then used to identify genes with consistently up- or downregulated expression across time points, and these genes were subjected to KEGG pathway enrichment analysis to identify potential drought resistance pathways. These findings elucidate the untapped genetic potential for drought resistance among mulberry germplasm resources in Southwest China, particularly in Yunnan Province.

## Materials and methods

### Plant materials and drought treatment

Yunsang-2 seedlings, provided by the Sericulture and Mulberry Research Institute, Yunnan Academy of Agricultural Sciences, were planted into pots in the greenhouse of Qujing Normal University, Qujing, Yunnan Province, China, during the summer season. The natural environmental conditions were as follows: the temperature ranged from 25 °C to 35 °C during the day and 18 °C to 25 °C at night, and the photoperiod was approximately 14 h light/10 h dark (natural daylight). Seedlings were grown in plastic pots (30 cm diameter, 40 cm height) filled with mixed soil of loess and nutrient soil at a ratio of 2:1 (v/v). Uniformly grown seedlings were selected for the drought stress experiments. To synchronize the seedling growth status for both the drought-stressed group (DS) and the control group (CK), irrigation was suspended for 7 consecutive days prior to the onset of the formal drought treatment. During these seven days, soil moisture gradually declined but remained sufficient to support normal seedling growth, and no visible drought symptoms were observed. The 8th day following the cessation of watering was defined as 0 days post-stress (DPS), representing the baseline condition for both groups before drought imposition began. From 0 DPS onward, no further irrigation was applied to the DS, whereas the CK was regularly watered to maintain normal soil moisture throughout the experimental period. Leaf samples were collected from both the DS and CK groups at 0, 3, 5, 7, 9, and 12 DPS. For the DS group, DPS indicates days after drought initiation; for the CK group, samples were taken on the corresponding calendar days (i.e., Days 0, 3, 5, 7, 9, and 12 after the start of the experiment). Leaf samples were collected from the 5th to 6th fully expanded leaves from the apex, which had reached their maximum leaf area, for all the physiological and transcriptomic analyses. Three independent biological replicates were performed for each treatment, with 10 seedlings per replicate. All the collected samples were immediately frozen in liquid nitrogen and stored at − 80 °C.

### Determination of the proline and soluble protein contents and the activity of catalase (CAT) and peroxidase (POD) in mulberry leaves.

The proline content was determined using the ninhydrin colorimetric method as described by Kamruzzaman et al. [33]. In brief, ninhydrin reagent (1.25 g of ninhydrin in 30 mL of glacial acetic acid and 20 mL of 6 mol/L phosphoric acid) and 3% sulfosalicylic acid (SA) were prepared fresh with a heat and dissolve method. Approximately 0.5 g of mulberry leaves (fresh weight, FW) was homogenized in 5 mL of 3% sulfosalicylic acid and extracted at 100 °C for 10 min. The supernatant (2 mL) was mixed with 2 mL each of glacial acetic acid and acidic ninhydrin and then heated at 100 °C for 30 min. After adding 4 mL of toluene and vortexing, the chromophore was separated by centrifugation (3000 rpm, 5 min, Eppendorf 5810R), and the absorbance was measured at 520 nm against a toluene blank. The proline content of each sample was determined on the basis of a standard curve and expressed as mg/g FW of the plant.

The soluble protein concentration was determined using a Coomassie brilliant blue assay kit (Cat# M1805A) according to the instruction manual. Similarly, the activities of CAT and POD were determined using a CAT activity detection kit (UV absorption method, M0103A) and a POD activity detection‌ kit (colorimetric assay, M0105A), which were provided by Michy Biotechnology Company (http://www.michybio.com, Suzhou, China). All procedures were performed strictly according to the manufacturer’s protocols. All the physiological measurements were performed using the same three biological replicates. Finally, the data were visualized and statistically analyzed using GraphPad Prism 9 software.

### RNA isolation, library construction, and RNA-Seq analysis

Total RNA was extracted from the leaf samples using an RNAsimple Kit (DP419; Tiangen, Beijing, China) following the manufacturer’s protocol. The RNA was quantified using a NanoDrop2000 (Thermo Scientific, Waltham, MA, USA). Agarose gel electrophoresis (1%) and an Agilent Bioanalyzer 2100 (Agilent Technologies) were used to assess RNA integrity. A total of 2 µg of RNA (RIN ≥ 7.0) from each sample was used for transcriptome library sequencing. Libraries were constructed using an Illumina TruSeq RNA Sample Preparation Kit (Illumina) following the manufacturer’s recommendations. A total of 15 libraries (three biological replicates for each of the five conditions: 0 DPS, 7 DPS CK, 7 DPS DS, 12 DPS CK, and 12 DPS DS) were sequenced using a NovaSeq™ XPlus sequencing platform with PE150 raw reads (Berry Genomics Co., Ltd., Beijing, China).

Clean data were obtained by removing reads from the raw data that contained adapter, poly-N, and low-quality reads using Trimmomatic software. Finally, the percentage GC content and Q30 were determined for the clean data, which were subsequently used for downstream analysis. Hence, the clean data were mapped to *M. notabilis* genomes [34] using HISAT2 (v2.1.0) software [35]. The transcriptomes of each sample were assembled on the basis of mapped reads and merged using StringTie software (v2.0), and the FPKM values were calculated using StringTie and Ballgown [36]. The count matrix was extracted using the prepDE.py script and subsequently analyzed for differentially expressed genes (DEGs) with the DESeq2 package in R (v4.4.2) [37]. The DEGs were identified using the following criteria: expression level (FPKM) > 1.0, false discovery rate (FDR) < 0.001, and |log_2_(fold change)| ≥1 for each pairwise comparison.

### Functional annotation and transcription factor analysis

The protein sequences of all the DEGs were extracted from the reference genome annotation and used for BLASTP (E-value = 1 × 10^− 5^) to find the best hit in *Arabidopsis thaliana*, along with its gene definition. DEGs sharing consistent expression profile trends were analyzed with a Venn diagram using VennPainter software. Gene Ontology (GO) enrichment analysis was performed using the clusterProfiler package in R (v4.4.2). A significance threshold of adjusted p-value < 0.05 was applied, and the Benjamini–Hochberg procedure was used for multiple-testing correction. Kyoto Encyclopedia of Genes and Genomes (KEGG) enrichment analysis was performed on the KOBAS 3.0 website [38]. The top 20 significant pathways according to the q value were visualized using R packages.

TF prediction and classification were performed in PlantTFdb (https://planttfdb.gao-lab.org). The expression profiles of the DEGs were visualized using a heatmap program in TBtools-II.

### Phylogenetic tree construction of mulberry ERF TFs and functional analysis in yeast cells

To elucidate the functional roles of TFs in the regulation of drought stress, we constructed a phylogenetic tree of the ERF subfamily, which typically contains one AP2 domain, and the 14th and 19th amino acids were A (Ala) and D (Asp), respectively [39]. In brief, the *A. thaliana* ERF subfamily in the AP2/ERF superfamily was retrieved from TAIR (www.arabidopsis.org) and used to search ERF TFs in Morus genome annotation files using the BLASTP program, with an E-value of 1 × 10^− 6^. The subject sequences were subsequently validated on the pfam (https://pfam.xfam.org) and SMART (https://smart.embl.de/) websites to identify the AP2 domain. Multiple sequence alignment was performed using MEGA11 software, and a neighbor-joining tree was constructed on the basis of the conserved AP2 domain, with a bootstrap value of 1000.

The coding sequence of the ‌*MnERF21* gene‌ was subsequently cloned from the leaf cDNA of ‌Yunsang-2. Using a double digestion approach, the sequence was cleaved with *the* restriction enzymes BamH*I* and EcoR*I* and then inserted into the ‌pYES2 vector‌ via the “In-Fusion” cloning method. After the correct sequence was confirmed by sequencing, the recombinant plasmid was transformed into the ‌INVSc1 yeast strain (PYTBio, Wuhan). Single positive clones were selected and serially diluted to optical densities with OD_600_ values of 0.2, 0.02, 0.002, and 0.0002. Yeast cells were then spotted onto ‌SG/-Ura (PYTBio) solid medium‌ containing varying concentrations of ‌sorbitol‌ to analyze the role of the gene in conferring ‌drought stress tolerance‌, with the empty plasmid used as a control.

### qRT‒PCR analysis

RNAs were extracted from leaf samples collected at 3, 7, 9 and 12 DPS. After its integrity was detected by agarose gel electrophoresis, 1 µg of RNA was reverse transcribed using EasyScript^®^ One-Step gDNA Removal and cDNA Synthesis SuperMix (AE311, TransGen). Real-time PCR was performed on a Roche 480 PCR system with PerfectStart^®^ Green qPCR SuperMix (AQ601-01, TransGen). The qPCR protocol was performed according to a previous study [40]. *MnActin*7 was used as a reference gene. The 2^−∆∆Ct^ method was used to calculate the relative expression of each gene, with three technical repetitions and three biological repetitions. Two-way ANOVA was used to determine the significance of the differences. The data are shown as the mean ± SD and were visualized using GraphPad Prism 9.3. The sequences of primers used in the present study are listed in Supplementary Table 5.

## Results

### Physiological changes and RNA-Seq results for Yunsang-2 under drought stress

**Fig. 1:**
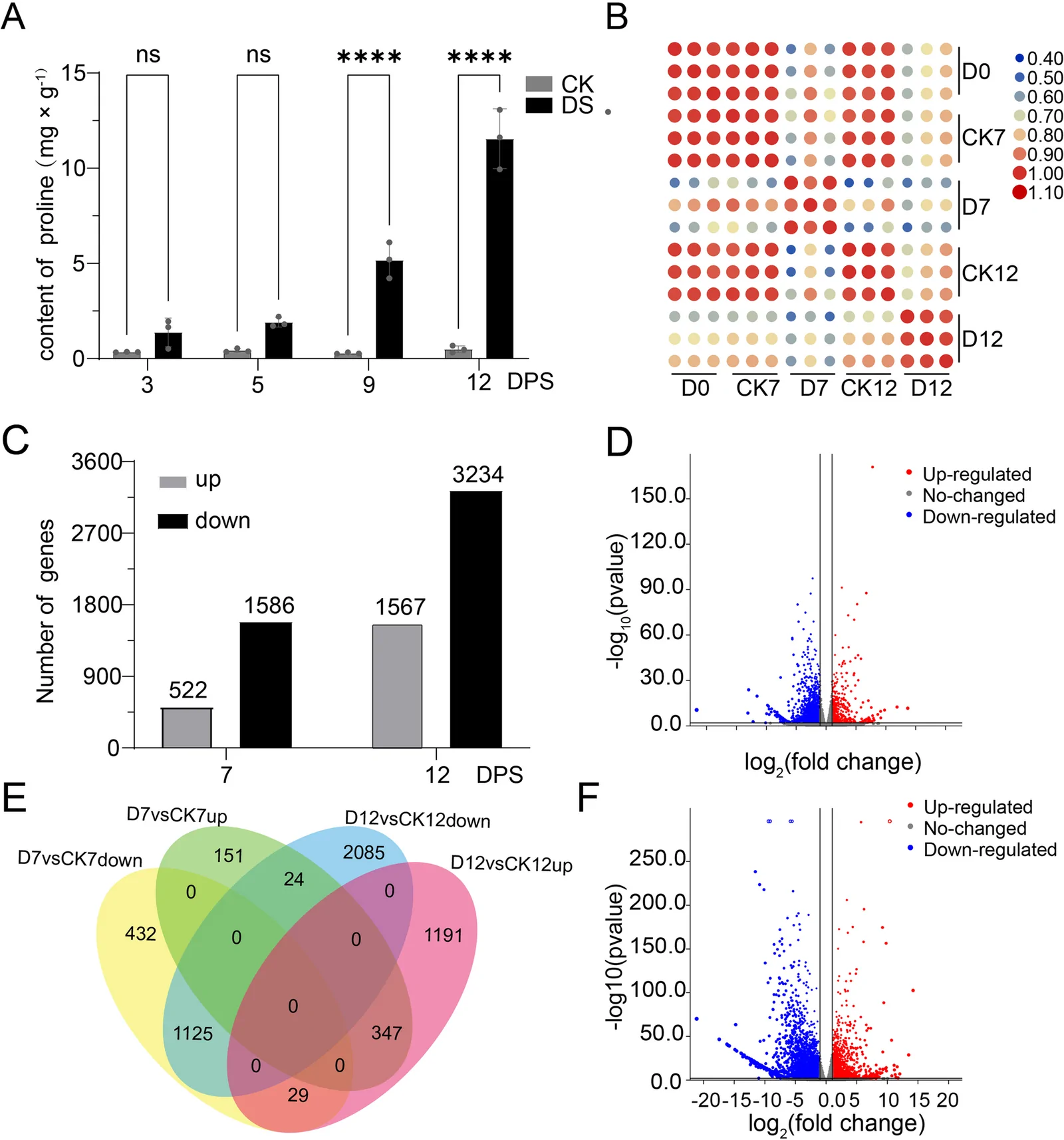
Overview of the results of the RNA-Seq experiment.‌ **A** Determination of the proline content in the leaves of Yunsang-2 seedlings at different time points under drought stress. **B** Pearson correlation analysis of biological replicates among RNA-Seq samples. **C** Bar plot showing the number of differentially expressed genes (DEGs) between the DS and CK groups at 7 and 12 DPS. **D** and **F** Volcano plots displaying DEGs at 7 and 12 DPS (red: upregulated; blue: downregulated). **E** Venn diagram showing counts of exclusive and shared DEGs across four differential gene subsets, including upregulated and downregulated genes derived from D7 vs. CK7 and D12 vs. CK12 comparisons

Following drought stress, Yunsang-2 seedlings exhibited symptoms of leaf wilting and reduced turgor pressure (Supplementary Fig. 1). The determination and analysis of proline content revealed a significant increase in proline content in Yunsang-2 leaves after 9 and 12 days of drought stress. (Fig. 1A). Soluble proteins are key substances, whereas CAT and peroxidase (POD) are key antioxidant enzymes synthesized by plants to increase stress resistance under abiotic stress conditions such as drought. Consequently, we measured the content of soluble proteins, along with the activities of CAT and POD. The results indicated that the soluble protein content significantly improved after 3 DPS. In contrast, the CAT and POD enzyme activities began to significantly increase until 7 DPS. All four physiological indices were highly significantly upregulated by 12 DPS. These results indicated that Yunsang-2 is a relatively drought-responsive cultivar.

Considering that CAT activity and POD activity significantly increased starting at 7 DPS (indicating an early oxidative stress response), whereas proline accumulation significantly increased only at 9 and 12 DPS (reflecting late osmotic adjustment), and together with the baseline measurement at 0 DPS, we selected leaf tissues at 0 DPS, 7 DPS (early response), and 12 DPS (late response) for RNA-Seq. After sequencing and data quality control, the Q30 values consistently exceeded 96%, and the GC content ranged between 46.1% and 47.3% (Supplementary Table 1), which is slightly higher than that reported in Li’s study [41]. Pearson correlation coefficients above 0.85 were significant between biological replicates, indicating reliable reproducibility (Fig. 1B). These results confirm that the data are suitable for subsequent analyses.

### Identification of potential drought-responsive functional pathways and genes

RNA-Seq was used for comparisons across three time points (0, 7, and 12 DPS) and two groups (CK and DS) in terms of both temporal and group dimensions. Differentially expressed genes (DEGs) among the temporal comparisons between “7 DPS and 0 DPS” and “12 DPS and 7 DPS” and group comparisons between “D7 and CK7” and “D12 and CK12” were identified (Supplementary Table 2). First, in the group comparisons at 7 DPS, 1586 and 522 genes were significantly up- and downregulated, respectively, in the drought group compared with those in the CK group. Similarly, at 12 DPS, 3234 and 1567 genes were significantly up- and downregulated, respectively, under drought stress. Venn diagram analysis revealed that 347 and 1125 genes were consistently up- and downregulated, respectively, at both 7 and 12 DPS. Furthermore, 29 genes were downregulated at 7 DPS but upregulated at 12 DPS, whereas 24 genes were upregulated at 7 DPS but downregulated at 12 DPS. These genes may play sequential and crucial roles in regulating plant life activities during drought stress in mulberry.

**Fig. 2:**
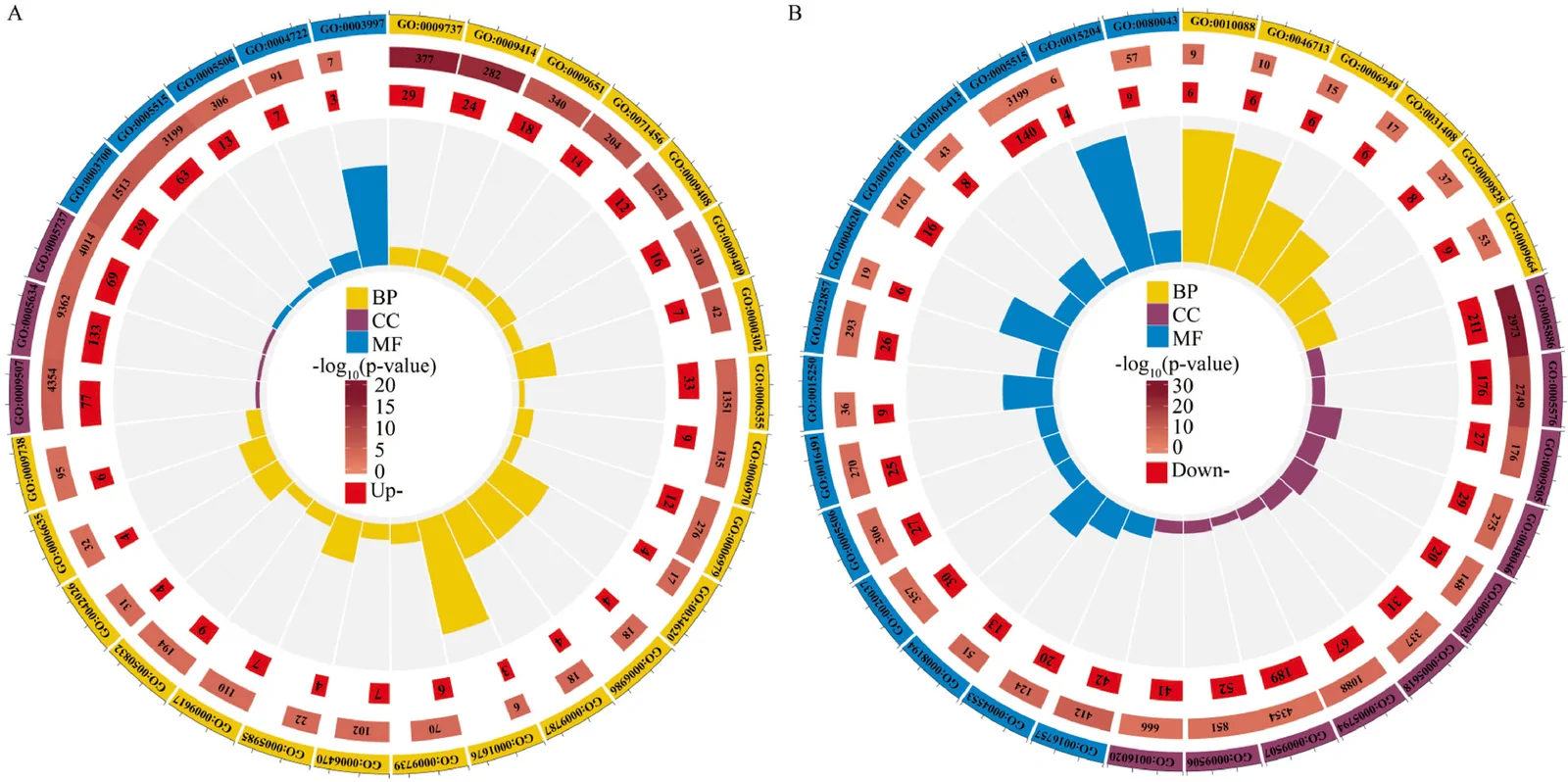
GO enrichment analysis of the 347 upregulated (**A**) and 1125 downregulated (**B**) genes consistently differentially expressed at both 7 and 12 DPS.‌ The top 30 significant terms are visualized and categorized as biological process, cellular component, and molecular function

To explore the function through which these genes regulate drought resistance, GO enrichment analysis was performed on the 347 genes whose expression was consistently upregulated and the 1125 genes whose expression was consistently downregulated (Fig. 2). The results revealed that the up- and downregulated genes were associated with the biological process (BP), cellular component (CC) and molecular function (MF) terms, and the top 30 significantly enriched terms were visualized on the basis of the p value. According to Figs. 2A and 22 of the 30 terms belonged to the BP category, of which the terms “response to abscisic acid” (GO:0009737) and “response to water deprivation” (GO:0009414) were the most significant, indicating that Yunsang-2 primarily enhanced its drought resistance through the ABA signaling pathway. Similarly, among downregulated genes, “plasma membrane” (GO:0005886), “extracellular region” (GO:0005576), and “plant-type cell wall” (GO:0009505) were the three most significantly enriched terms, suggesting a possible association between the repression of cell wall development and the drought response in Yunsang-2.

**Fig. 3:**
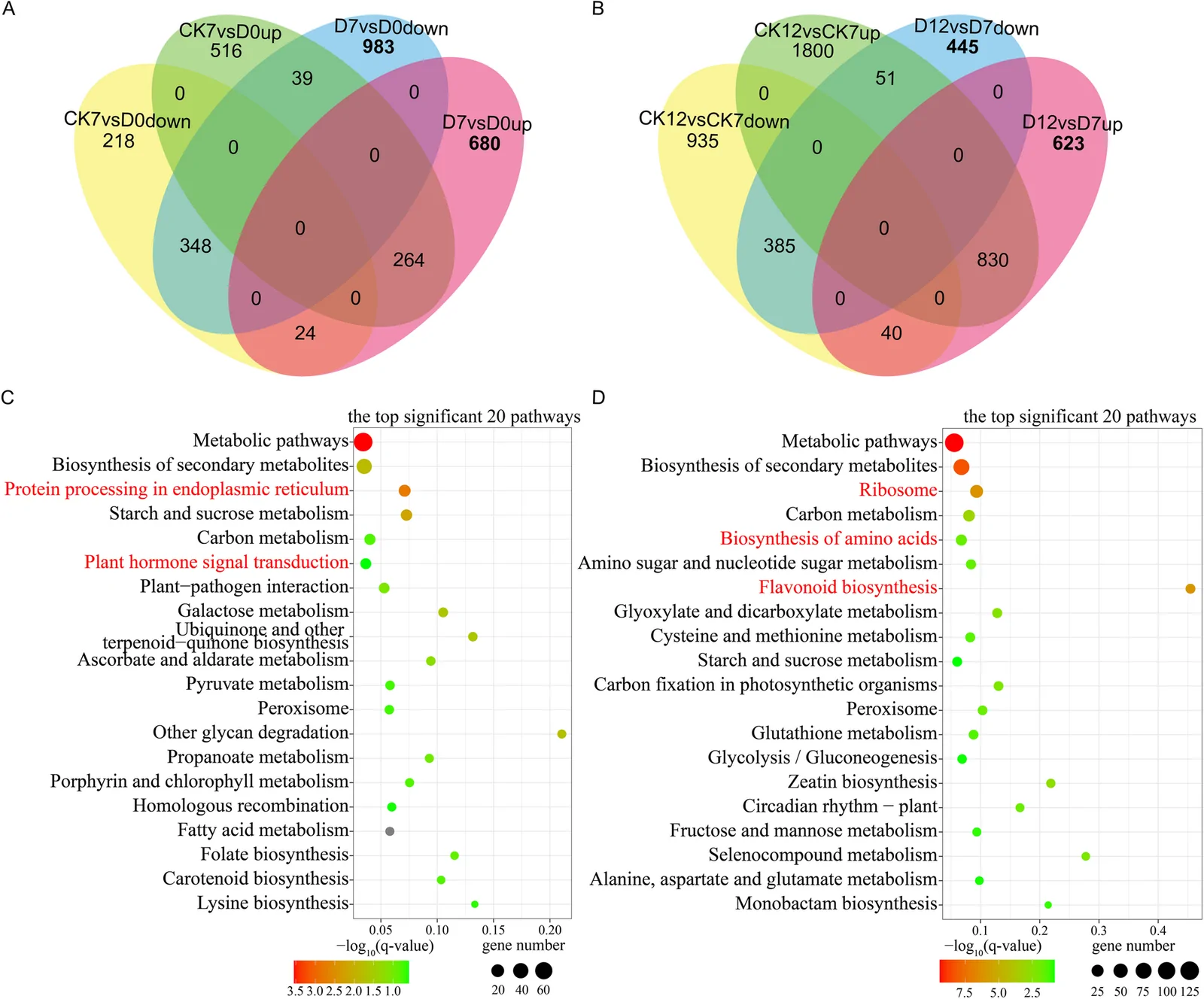
Screening of potential drought-responsive functional genes.‌ **A** Number of DEGs exclusive to each horizontal comparison (between D7 and D0) and shared among the four comparisons. **B** Number of DEGs exclusive to each horizontal comparison (between 12 and 7) and shared among the four comparisons. **C** and **D** KEGG enrichment analysis of the 680 and 983 genes, of which the expression significantly changed after drought treatment in D7 vs. D0

**Fig. 4:**
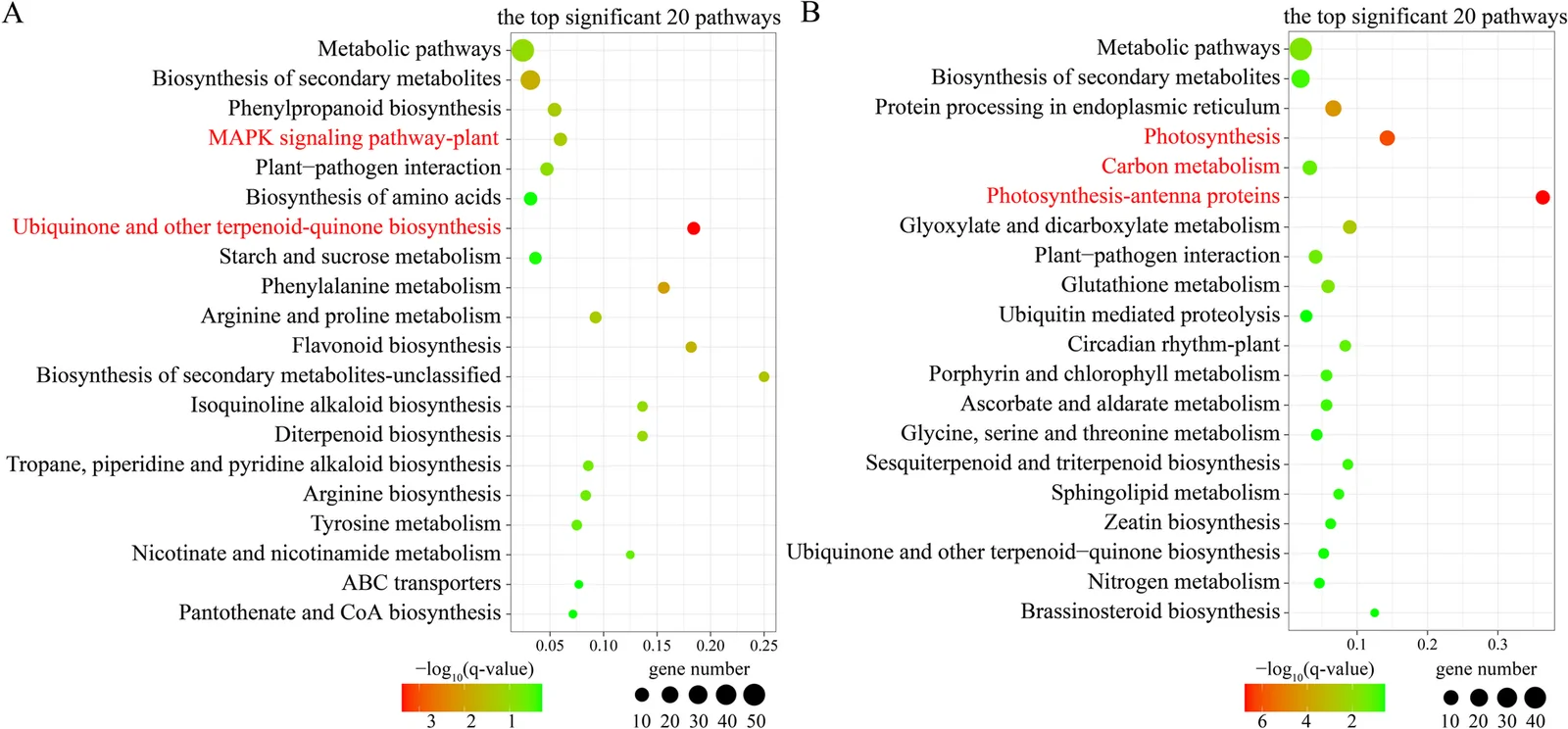
KEGG enrichment analysis of the 623 up- and 445 downregulated genes in the comparison of D12 vs. D7 after drought treatment

To determine whether the DEGs identified in the temporal comparisons were involved in either general plant growth processes or specific drought responses, Venn diagram analysis was conducted (Fig. 3A and B). The results revealed 680 and 983 genes that were specifically up- and downregulated, respectively, in the drought group at 7 DPS compared with those at 0 DPS. KEGG enrichment analysis revealed that these upregulated genes were enriched in the “protein processing in the endoplasmic reticulum” and “plant hormone signal transduction” pathways, whereas the 983 downregulated genes were enriched in the “ribosome”, “biosynthesis of amino acids”, and “flavonoid biosynthesis” pathways. Similarly, 623 and 445 genes were specifically up- and downregulated, respectively, in the drought group at 12 DPS compared with those at 7 DPS. KEGG analysis revealed that these genes whose expression was temporally upregulated (12 vs. 7 DPS) were enriched mainly in “ubiquinone and other terpenoid–quinone biosynthesis” and “MAPK signaling pathway - plant”, whereas the genes whose expression was downregulated were enriched in “carbon metabolism” and “photosynthesis and its antenna proteins” (Fig. 4). We acknowledge that the strict statistical threshold (FDR < 0.001) used for DEG identification may have excluded some relevant genes with lower expression changes. These results suggest that Yunsang-2, as a putatively drought-tolerant mulberry cultivar, regulates drought resistance during the early stages of stress by modulating plant hormone signaling and reducing flavonoid biosynthesis, amino acid synthesis, and amino sugar and nucleotide sugar metabolism. Under prolonged and severe drought stress, it maintains basic life activities by increasing the biosynthesis of phenylpropanoids, ubiquinone and other terpenoid-quinones while suppressing photosynthesis.

To comprehensively analyze the reason for drought resistance in Yunsang-2, the expression of genes enriched in plant hormone signal transduction was examined and is listed in Supplementary Table 4. These genes were enriched in the ABA, auxin, cytokinin, GA and BR signaling pathways. Twelve DEGs were enriched in the ABA signaling pathway. In this pathway, the expression of three ABA receptor-encoding genes was downregulated, whereas the expression of three PP2C genes, one SnRK gene, and two ABF genes was significantly upregulated, indicating that ABA is a crucial regulatory pathway according to the transcriptome data. Fourteen auxin genes, including IAA, SAUR-like, AUX/IAA, and ARF and six cytokinin-encoded response regulators (ARRs) and histidine-containing phosphotransfer factors (AHPs) were subsequently differentially expressed under drought stress. Additionally, two upregulated DEGs, encoding a GRAS TF and a phytochrome-interacting factor (PIF3) that increases drought resistance in rice by activating the transcription of PP2C and DREB2A [42], may play roles in drought stress. *AtBSK5* is inducible under drought and ABA exposure, and its *bsk5* loss-of-function mutant is sensitive to salinity and ABA [43, 44]. In the present study, the expression of two BSK and BAK transcripts increased at 12 DPS, suggesting that the BR signal contributes to drought resistance in mulberry. These findings pave the way for further functional analysis of phytohormone-mediated regulatory mechanisms of drought resistance in mulberry.

### Transcription factor analysis

**Fig. 5:**
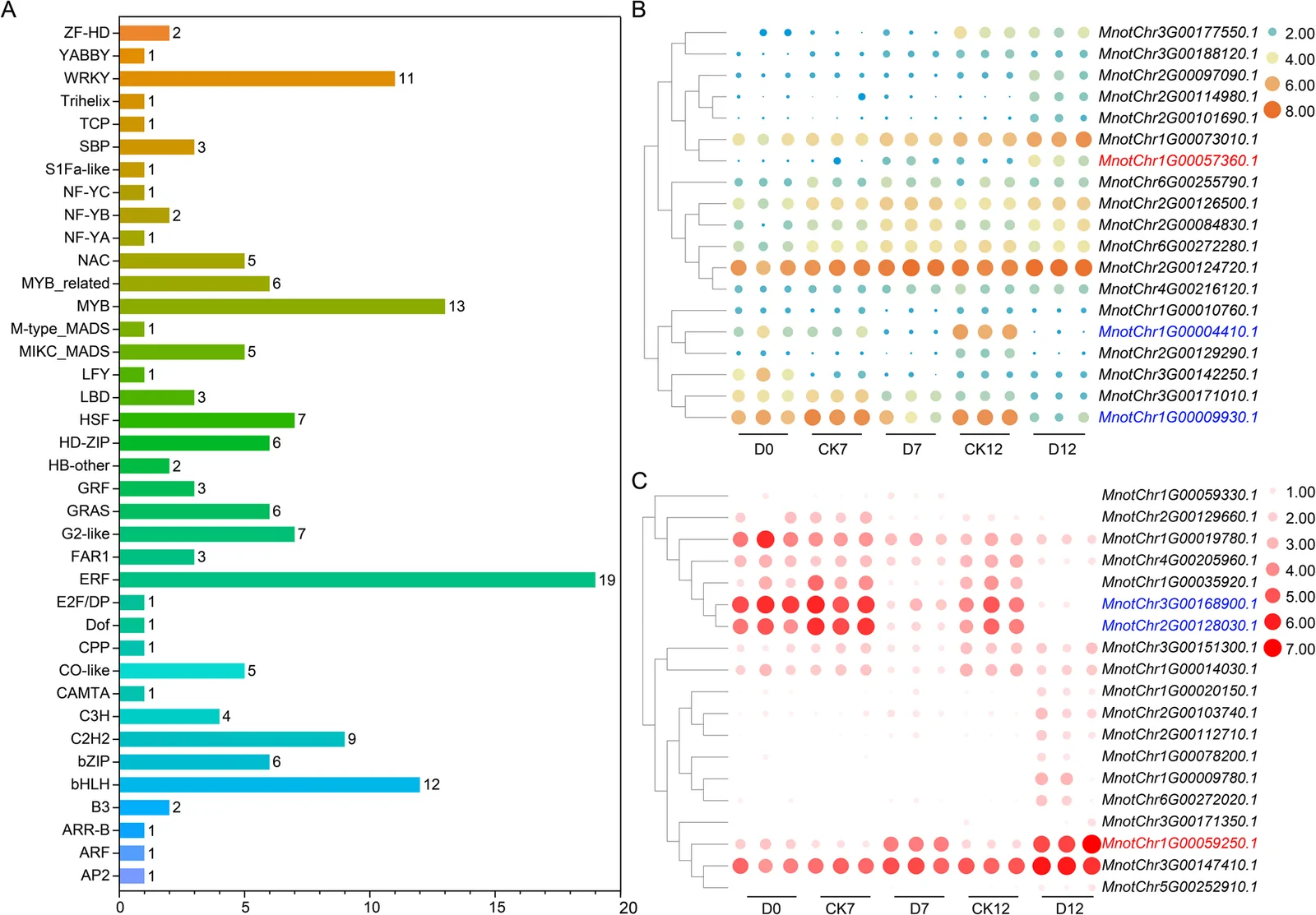
Transcription factor (TF) analysis among drought-responsive candidates.‌ **A **Classification of 156 identified TFs. **B** Heatmap of expression profiles for 19 ERF family TFs in 15 samples. **C** Heatmap of expression profiles for 19 MYB/MYB-related TFs in 15 samples

Transcription factors (TFs) are crucial regulators that modulate the expression of downstream genes to synthesize protective compounds such as proline and CAT in response to drought stress signals [45, 46]. Among the 2253 DEGs specifically attributed to drought stress, 156 were identified as TFs. Bar plot analysis categorized these TFs into 38 families (Fig. 5A), including 19 ERF, 19 MYB (including 6 MYB-related), 12 bHLH, and 11 WRKY TFs. To visualize the expression patterns of the two most abundant TF families, ERF and MYB, heatmaps and clustering analysis were generated to reveal the expression levels of these differentially expressed TFs across all the samples (Fig. 5B and C). For example, the ERF TF *MnotChr1G00057360.1* was upregulated at 7 and 12 DPS, whereas *MnotChr1G00004410.1* and *MnotChr1G00009930.1* were significantly downregulated. Among the MYB TFs, *MnotChr3G00168900.1* and *MnotChr2G00128030.1* were significantly downregulated under drought stress, whereas *MnotChr1G00059250.1* was significantly upregulated. Notably, the expression trends of *MnotChr1G00059250.1* and *MnotChr1G00057360.1* (renamed MnERF21) were concordant with the subsequent qPCR validation results (Fig. 7), reinforcing the reliability of the RNA-Seq data. These findings indicate that these TFs likely play critical roles in regulating the drought resistance process in mulberry.

**Fig. 6:**
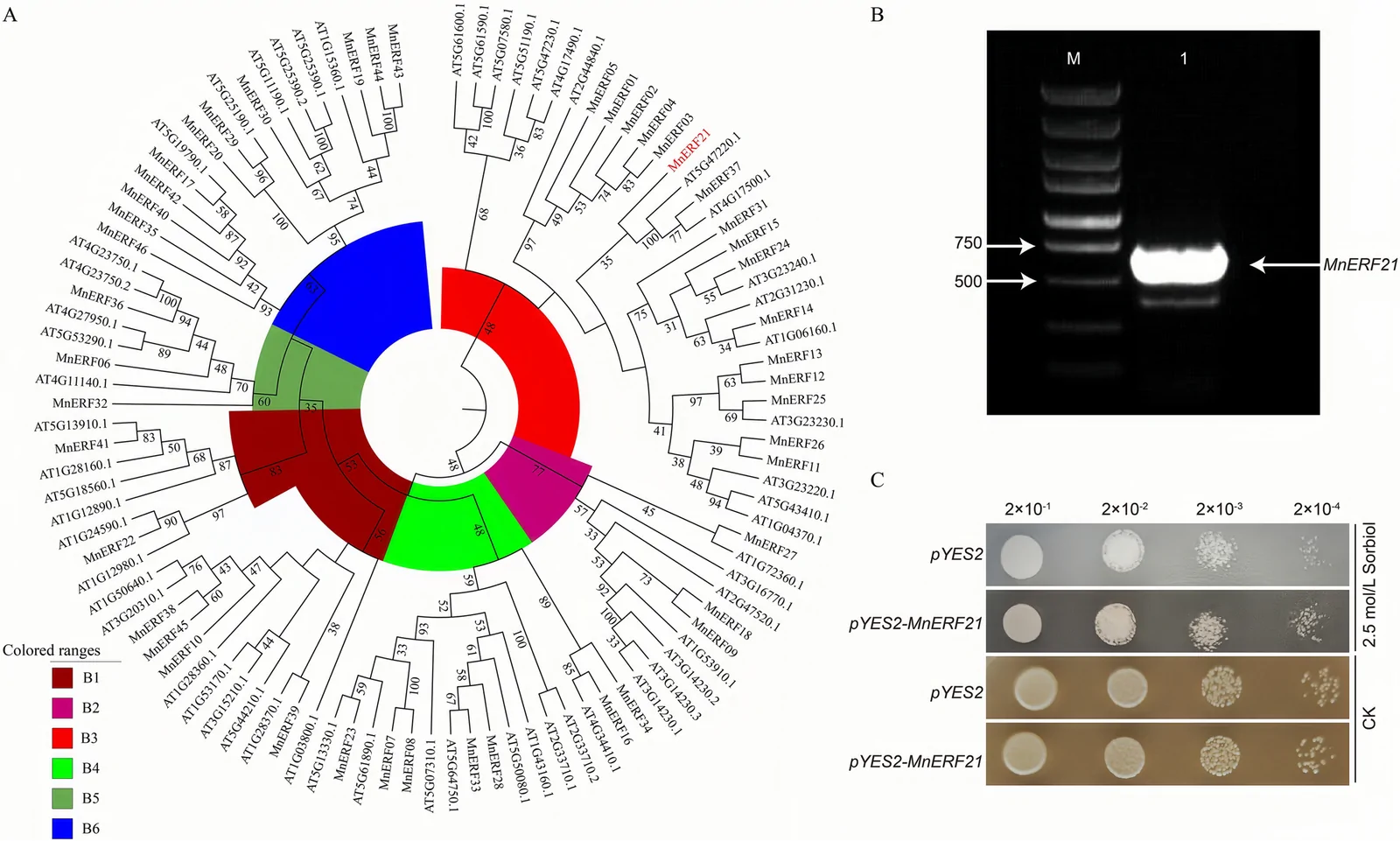
Phylogenetic relationships of the ERF subfamily in *M. alba* and *A. thaliana*. In addition, a functional analysis of the effects of *MnERF21* on drought resistance in yeast cells was performed. **A** Forty-six ERFs were divided into six groups on the basis of *A. thaliana* ERF expression. **B** Agarose gel electrophoresis of the *MnERF21* gene clone. **C** Drought resistance analysis of *pYES2-MnERF21* on SG/-Ura plates

To further analyze the drought resistance function of the TFs, we constructed a phylogenetic tree of the ERF subfamily and renamed *MnotChr1G00057360.1 MnERF21*. Phylogenetic analysis revealed that MnERF21 is closely related to AT5G47220.1 and AT4G17500.1 in the B3 subgroup (Fig. 6A). Since its expression could be induced by drought stress, we transformed the coding sequence of *MnERF21* in yeast cells. The results of the drought simulation experiment revealed that compared with the control, *pYES2-MnERF21* significantly enhanced drought resistance under 2.5 mol·L⁻¹ sorbitol stress in SG/-Ura medium (Fig. 6B, C). These findings suggest that ‌MnERF21 may play a role in drought resistance in mulberry‌ and support the finding that our transcriptome analysis led to the identification of a set of candidate genes for drought resistance.‌.

### qRT‒PCR validation of differentially expressed genes

**Fig. 7:**
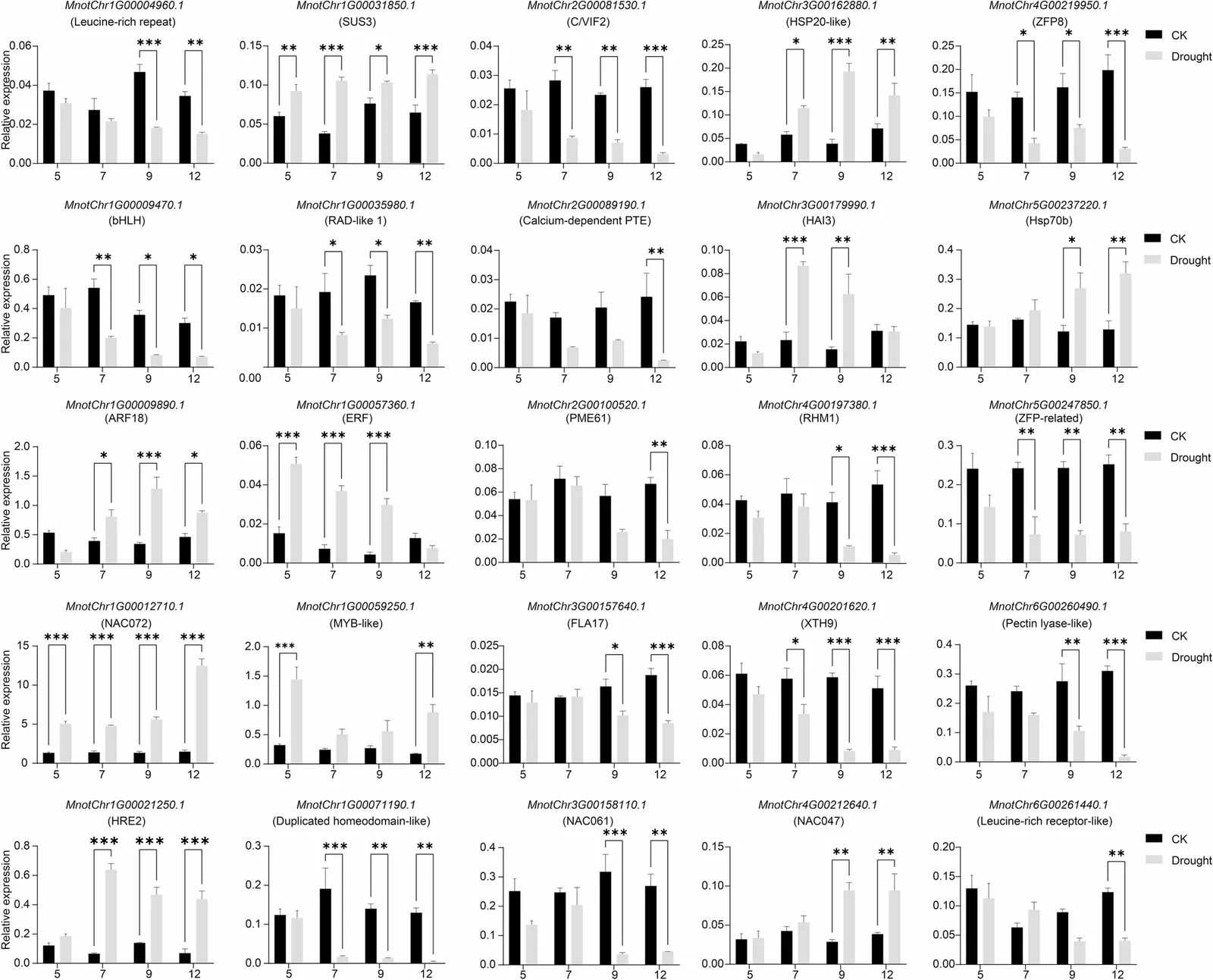
qRT‒PCR validation of the RNA-Seq data. A total of 25 genes were randomly selected for qRT‒PCR. The *MnActin7* gene was used as a reference gene, and the data are shown as the mean ± SD. Two-way ANOVA with Tukey's post hoc test was used to determine the significance of the differences

To validate the reliability of the transcriptome data, we randomly selected 25 differentially expressed genes (DEGs) for qPCR analysis (Fig. 7). These genes encode a diverse range of proteins, including leucine-rich repeat proteins, sucrose synthases, heat shock proteins, and transcription factors from the NAC, MYB, bHLH, and ARF families, representing various functional categories and expression levels observed in our RNA-Seq dataset. The qPCR results strongly agreed with the RNA-Seq expression profiles for the vast majority of the selected genes across different time points under drought stress. For instance, key transcription factors such as *MnERF21* (*MnotChr1G00057360.1*), *MnotChr1G00059250.1* (MYB-like), and *MnotChr4G00212640.1* (NAC047) were consistently upregulated in both datasets, whereas genes such as *MnotChr3G00158110.1* (NAC061) and cell wall development-related genes such as *MnotChr6G00260490.1* (pectin lyase-like) and *MnotChr4G00201620.1* (XTH9) were confirmed to be downregulated.

To quantitatively assess the consistency between the two methods, we performed a linear regression analysis to compare the log₂ fold change (log₂FC) values from RNA-Seq with the log₂(2^⁻ΔΔCt^) values from qPCR for all 25 genes at their respective time points of significant differential expression (Table S3). The analysis revealed a highly significant positive correlation (R² = 0.82, *p* < 0.001), providing robust statistical evidence for the technical accuracy and reproducibility of our transcriptome sequencing data.

This successful validation not only confirms the high quality of our RNA-Seq dataset but also reinforces the credibility of the identified DEGs and the subsequent biological insights derived from them, such as the pivotal role of specific TF families and hormone signaling pathways in the drought response of Yunsang-2.

## Discussion

Advances in sequencing technologies have established transcriptomic analysis as a mainstream approach for functional gene discovery [47]. For instance, integrated multiomics strategies—including miRNA sequencing, metabolomics, and noncoding RNA profiling—have been employed in *M. alba* cv. Yu-711 to decipher drought-responsive gene expression and metabolic reprogramming [1, 48, 49]. However, Yu-711 is located primarily in low-altitude, high-precipitation regions such as Zhejiang and Jiangsu Provinces. In Yunnan Province, Southwest China, ecologically adapted cultivars from the Yunsang cultivar (developed by the Sericulture and Apiculture Research Institute, Yunnan Academy of Agricultural Sciences) dominate local cultivation. Notably, these regionally critical germplasms remain underexplored in molecular drought studies.

### Molecular basis of drought tolerance in Yunsang-2

In this study, plant physiological techniques and transcriptomics were used to elucidate the transcriptomic response characteristics of Yunsang-2 under drought stress. Following drought treatment, samples collected at multiple time points were analyzed for their proline content, soluble protein content, and CAT and POD activities. Given that proline and POD serve as critical downstream effectors in plant drought adaptation mechanisms, the observed early accumulation of soluble proteins (3 DPS) followed by a delayed proline surge (9 DPS) and antioxidant enzyme activation (CAT/POD at 7 DPS) suggests a tiered defense strategy (Figure S1B–D). For example, the proline synthase gene [50] P5CS (*MnotChr1G00072480.1*) was not significantly upregulated on Day 7 but was significantly upregulated on Day 12. Unlike in drought-sensitive species, in which proline spikes rapidly (e.g., *A. thaliana* and *Maize* [51]), the delayed proline response of Yunsang-2 may reflect its inherent tolerance, reserving protective compounds for severe stress—a trait advantageous for prolonged dry seasons in Yunnan. On the basis of these physiological results, samples at 7 and 12 DPS were selected for transcriptome sequencing.

Transcriptional reprogramming represents a fundamental molecular adaptation to abiotic stress, in which genes are differentially expressed under the regulation of plant hormones and TFs to regulate the transcription of downstream genes and synthesize osmolytes essential for maintaining cellular homeostasis. ABA signaling-induced drought resistance is a core transcriptional regulatory mechanism in plant drought tolerance, which is consistent with the findings of previous studies [41]. However, this study revealed that in addition to the ABA pathway, the auxin, cytokinin, GA and BR signaling pathways may contribute to increasing drought tolerance in Yunsang-2 mulberry (Table S4). With respect to the GA pathway, a recent study demonstrated that PIF3 can activate the auxin signaling gene YUC8 and upregulate the cell wall expansion-related gene expansin (EXPA) in *Populus* [52]. In our study, the increased transcriptional level of ‌PIF3‌ may have resulted from the significant downregulation of the expression of ‌GRAS transcription factors‌, which normally promote cell division and development‌. These pathways likely improve drought resistance by altering the development of the cell wall and promoting leaf tissue cell development. For instance, the results of qRT‒PCR demonstrated that the expression of genes encoding *pectin methylesterase* (*MnotChr2G00100520.1*), *pectin lyase* (*MnotChr6G00260490.1*) and x*yloglucan endotransglucosylase* (*MnotChr4G00201620.1*), which are associated with cell wall development, significantly decreased under drought stress (Fig. 7). In addition, PME can be inducible by ABA to alter cell wall properties and influence stomatal behavior [53].

Our RNA-Seq analysis revealed that ERF, MYB and bHLH TFs constituted the most abundant classes among the drought-responsive TFs in Yunsang-2. Through Venn analysis, potential drought resistance-related TFs were discovered, among which several TFs have been shown to participate in the response to the abiotic stress caused by drought. For instance, a bZIP family TF (*MnotChr2G00130410.1*) encoding an ABA-responsive element binding factor (ABF) was upregulated at both 7 and 12 DPS compared with that in the CK condition. Its homolog, *GhABF3*, in upland cotton (*Gossypium hirsutum*) positively regulates drought tolerance [54]. Additionally, *MnotChr1G00009930.1* encodes the MnDREB4A protein, which has been reported to increase drought tolerance in tobacco [55]. However, in the present study, its expression was significantly downregulated following drought treatment. This divergence suggests that Yunsang-2 may employ unique transcriptional regulatory mechanisms distinct from those of previously studied models.

### Potential role of MnERF21 in mulberry drought tolerance

In recent decades, ERF TFs have been shown to be involved in the response to abiotic stresses such as drought and salt [56–58]. StERF79 enhances drought resistance by activating downstream late embryogenesis abundant (LEA) genes to increase the activity of the SOD and POD enzymes and proline content [59]. Interestingly, the genes *MnotChr1G00031700.1* and *MnotChr2G00110430.1*, which encode LEA3 proteins, were upregulated in the “D12 vs. CK12” comparison and may mediate tolerance to oxidative stress. They may also play key roles in adapting to drought environments [56]. *NtERF172*, induced by drought and ABA, directly binds to the promoter of NtCAT and enhances drought resistance by increasing CAT activity and mediating H_2_O_2_ homeostasis [58]. Compared with the WT, the overexpression of GmAP2/ERF144 in soybean resulted in increased drought resistance [57].

RNA-Seq experiments revealed that a large number of ERF TFs were differentially expressed under drought stress. We visualized the expression profiles of these ERF TFs (Fig. 5). A genome-wide analysis of *Morus* ERFs revealed that 46 ERF members can be divided into six groups on the basis of the ERFs of *A. thaliana*. Heterologous expression of *MnERF21* in yeast cells revealed that it may participate in increasing drought resistance (Fig. 6). However, further studies are needed to elucidate the drought resistance mechanism of *MnERF21* and other candidate DEGs. Therefore, subsequent research will focus on the functional characterization of these candidate DEGs and the elucidation of their drought-responsive transcriptional regulatory mechanisms, providing deeper molecular insights into drought tolerance mechanisms in mulberry.

## Conclusion

**Fig. 8:**
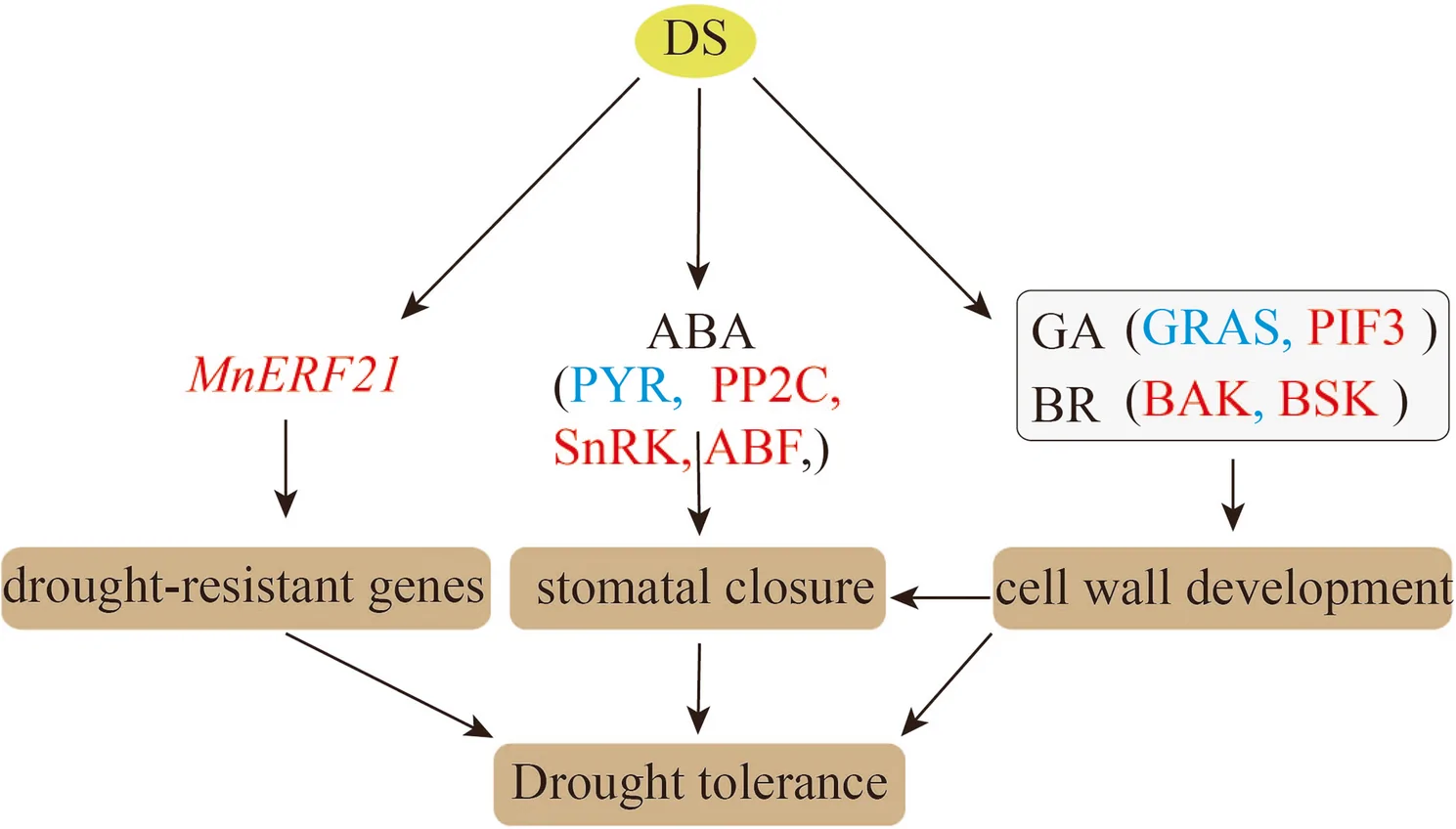
A simple model for elucidating the foundation of drought tolerance in Yunsang-2. Red and green indicate genes whose expression was up- and downregulated, respectively

In this study, we performed RNA-Seq and physiological detection on Yunsang-2 leaf tissue under drought stress. Proline, POD, and CAT are the compounds involved in maintaining life in plants. ‌The ABA signaling pathway serves as the core drought resistance mechanism in mulberry, while the GA and BR signaling pathways may also contribute to drought tolerance. Furthermore, our preliminary yeast-based validation suggests that MnERF21 may contribute to drought tolerance in mulberry, making it a candidate positive regulator that requires further in planta functional verification (Fig. 8).

## Supplementary Information

Supplementary Material 1: Figure S2. Multiple alignment sequences of the AP2 domain from ERF proteins identified from *M. alba.*

Supplementary Material 2: Table S1. Overview of the transcriptome sequencing data.

Supplementary Material 3: Table S2. FPKM values of all DEGs.

Supplementary Material 4: Table S3. RNA-Seq and qRT‒PCR data used for linear regression.

Supplementary Material 5: Table S4. List of DEGs enriched in the plant hormone signaling pathway.

Supplementary Material 6: Table S5. Primers for qPCR in the present study.

Supplementary Material 7: Figure S3. The original images and source of Figure 6-B. A. Agarose gel electrophoresis showing the initial cloning of the MnERF21 gene from a cDNA library. B. Re-electrophoresis of the *MnERF21 *gene alongside several other cloned genes on a single agarose gel for visual clarity and presentation purposes.

Supplementary Material 8: Figure S1. Drought treatment experiment of M. alba cv. Yunsang-2. (A) Phenotype of seedlings at 9 DPS. (B, C, D) Physiological indices: soluble protein content (B) and catalase (CAT, C) and peroxidase (POD, D) activities in leaves at 3, 7 and 12 DPS (n=3 biological replicates).

## Data Availability

RNA-Seq data were deposited into the National Genomics Data Center (NGDC) under the BioProject archive (PRJCA043810) and GSA accession number CRA028403 and are publicly available at the following URL: https://ngdc.cncb.ac.cn/gsa/browse/CRA028403.

## Abbreviations

ABA: Abscisic acid
ABF: ABA-responsive element-binding factor
BR: Brassinosteroid
BSK: BR signaling kinase
CAT: Catalase
CK: Control group
DEG: Differentially expressed gene
DPS: Days post-stress
DS: Drought-stress group
FDR: False discovery rate
FPKM: Fragments per kilobase of transcript per million mapped reads
FW: Fresh weight
GA: Gibberellin
GO: Gene Ontology
KEGG: Kyoto Encyclopedia of Genes and Genomes
LEA: Late embryogenesis abundant
MAPK: Mitogen-activated protein kinase
PIF3: Phytochrome-interacting factor 3
POD: Peroxidase
PYR: Pyrabactin resistance
qRT‒PCR: Quantitative real-time polymerase chain reaction
RIN: RNA integrity number
TF: Transcription factor
