# Supplementary material for: Comprehensive RNA-Seq analysis revealed molecular pathways and genes associated with drought tolerance in Morus alba cv. Yunsang-2
Source: BMC Plant Biol. 2026 Jul 15;26:1730. doi: 10.1186/s12870-026-09479-6 (PMC13651198; doi:10.1186/s12870-026-09479-6)
Supplement: Supplementary file 1 [file 12870_2026_9479_MOESM7_ESM.pdf]

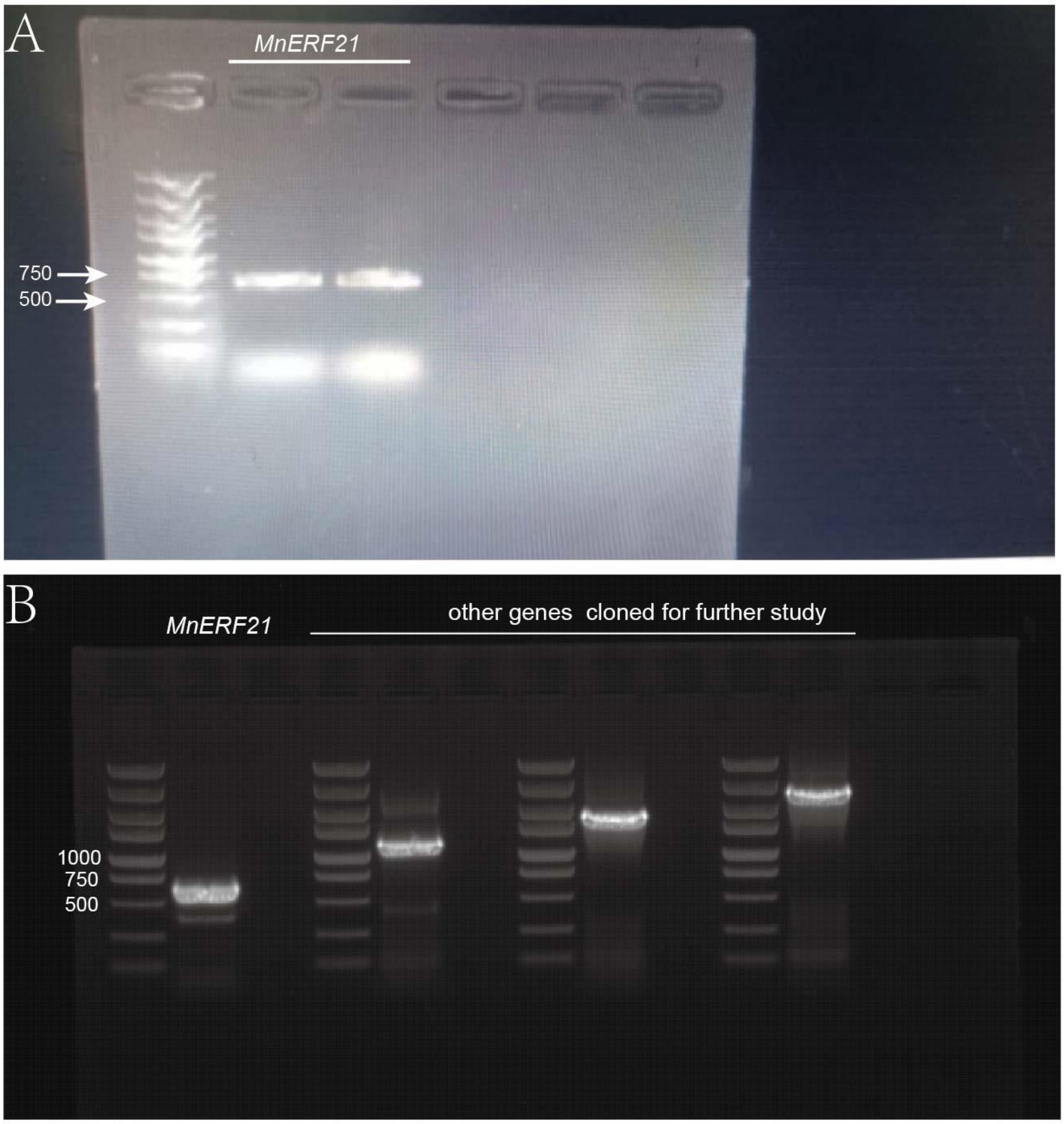

Figure S3 The original gel images and source of Figure 6-B.

A. Agarose gel electrophoresis showing the initial cloning of the MnERF21 gene from a cDNA library.

B. Re-electrophoresis of the *MnERF21* gene alongside several other cloned genes on a single agarose gel for visual clarity and presentation purposes.
